# NMR metabolomics assessment of neural commitment of human dental pulp-derived stem cells

**DOI:** 10.3389/fmolb.2026.1735166

**Published:** 2026-05-22

**Authors:** Mattea Chirico, Maria Elena Pisanu, Emanuela Mari, Valeria Manganelli, Fanny Pulcini, Loreto Lancia, Rita Di Benedetto, Simona Delle Monache, Egidio Iorio, Vincenzo Mattei

**Affiliations:** 1 High Resolution NMR Unit, Core Facilities, Istituto Superiore di Sanità, Roma, Italy; 2 Department of Life Science, Health and Health Professions, Link Campus University, Roma, Italy; 3 Department of Experimental Medicine, “Sapienza” University of Roma, Roma, Italy; 4 Department of Biotechnological and Applied Clinical Sciences, University of L'Aquila, L'Aquila, Italy; 5 Department of Food Safety, Nutrition and Veterinary Public Health, Istituto Superiore di Sanità, Roma, Italy

**Keywords:** ^1^H NMR, amino acid metabolism, dental pulp stem cells, metabolomics, neural commitment

## Abstract

**Background:**

Mesenchymal stem cells, particularly those derived from dental pulp cells (DPSCs), hold promising potential for neuro-regenerative therapies due to their multipotency and accessibility. Neural differentiation is closely linked to cellular metabolic reprogramming, yet the specific metabolic shifts alterations involved in DPSC neurogenesis remain underexplored.

**Methods:**

Neural commitment was induced by 14 days of differentiation with EGF and bFGF. Untargeted proton nuclear magnetic resonance (^1^H NMR) metabolomics was performed to investigate the metabolic alterations occurring during the neural commitment of human DPSCs cells.

**Results:**

Following 14 days of differentiation with EGF and bFGF, DPSCs exhibited a marked decrease in mesenchymal markers (CD44, CD90, CD105) and an increase in neural markers (β3-tubulin, NFH), alongside morphological changes toward a neuron-like phenotype. Metabolomics analysis revealed changes in metabolite levels, including increased aspartic acid and phosphocholine and reduced alanine, glutamate, and myo-inositol. Exploratory lipid analyses, suggested increased fatty acid and triacylglycerol content together with a higher PUFA/MUFA ratio. Pathway enrichment analyses highlighted amino acid metabolism and phosphoinositide signaling as potentially relevant. Understanding these changes enhances our knowledge of stem cell differentiation and supports the therapeutic potential of DPSCs in neuro-regenerative medicine.

**Conclusion:**

These findings provide a descriptive metabolic characterization of DPSC neural commitment and identify candidate metabolite changes associated with early neural differentiation. While the results support the occurrence of metabolic remodelling during neural induction, further studies integrating functional and flux-based approaches are required to define the biological significance of these alterations.

## 1 Introduction

Mesenchymal stem cells (MSCs) have gained substantial attention in regenerative medicine due to their potential to treat a wide range of diseases, particularly neurodegenerative disorders (Mattei and Delle Monache, 2024). Although tissue regeneration and stem cell functions are known to be regulated by cell metabolism, it remains unclear whether unique metabolic mechanisms exist that specifically govern stem cell activity. A review by Corbin and coworkers highlighted that stem cells possess a distinct metabolic signature, separate from that of more restricted progenitors, and that metabolic changes play a key role in regulating tissue homeostasis and regeneration (Corbin et al., 2022). Pluripotent stem cells (PSCs), embryonic stem cells (ESCs), and somatic stem cells (SSCs) have been considered primarily glycolytic for production of ATP (Rafalski et al., 2012). Glycolysis has been shown to support stem cell function not only through ATP production but also by supplying key biosynthetic intermediates-such as lipids, amino acids, and hexosamines needed for cell proliferation and survival (Zhang et al., 2012). Neural stem cells (NSCs), like other stem cell types, are also predominantly glycolytic in their undifferentiated state, shifting towards oxidative phosphorylation (OXPHOS) as they differentiate (Lange et al., 2016). Lange et al. demonstrated that proliferative NSCs derived from the embryonic cortex secrete significant amounts of lactate, which is markedly reduced upon differentiation, indicating a metabolic shift toward mitochondrial respiration (Dong et al., 2022). The advent of omics technologies has greatly expanded our capacity to study cellular metabolism. Understanding the metabolic basis of neural differentiation is crucial for manipulating stem cell behavior for therapeutic purposes. In particular, nuclear magnetic resonance (NMR) metabolomics offers a powerful method for real-time analysis of key metabolic pathways-including glycolysis, the tricarboxylic acid (TCA) cycle, and amino acid metabolism-during cellular reprogramming and differentiation. This makes NMR metabolomics especially valuable for identifying metabolic markers predictive of successful regenerative outcomes (Bispo et al., 2025). Dental pulp-derived mesenchymal stem cells (DPSCs) represent a subpopulation of adult stem cells isolated from tooth pulp, both deciduous and permanent (Martellucci et al., 2019a). These cells possess self-renewal capacity, multipotent differentiation potential, and immunomodulatory properties (Nuti et al., 2016). Compared to other sources of mesenchymal stem cells, such as bone marrow or adipose tissue, DPSCs are more easily accessible in a less invasive way. In addition, dental pulp is a source that is obtained with fewer ethical or legal problems compared to other procedures (Al Madhoun et al., 2021). DPSCs were isolated and characterized by Gronthos for the first time (Gronthos et al., 2000). Subsequently due to their favorable characteristics, they have gained considerable success, leading many researchers over the years to improve their isolation, characterization, differentiation and conservation procedures (Gronthos et al., 2000; Tirino et al., 2012; Rodas-Junco and Villicaña, 2017). They are identified through specific surface markers (e.g., CD90, CD73, CD105) and are distinguished by their ability to adhere to the substrate in culture (Dominici et al., 2006). In addition to being able to differentiate towards the mesodermal line, they also show the ability to transdifferentiate into ectodermal and endodermal lines (Yin et al., 2021). For these reasons, they are considered promising for applications in regenerative medicine and tissue engineering (Santilli et al., 2022; Ratajczak et al., 2016) particularly for the treatment of dental, bone, cartilage, and neurological pathologies (Hu et al., 2018; Martellucci et al., 2019b; Mortada and Mortada, 2018). Different studies have shown that DPSCs can also differentiate into neuron-like cells (Tatullo et al., 2015) and express a typical neural markers as nestin, microtubule-associated protein 2 (MAP2), neurofilament (NFH) and β3-tubulin (Darvishi et al., 2021; Pardo-Rodríguez et al., 2025). DPSC neural commitment has been demonstrated in several *in vitro* studies using different approaches and methods. Several growth factors are used to induce neural differentiation in DPSCs, such as epidermal growth factor (EGF), basic fibroblast growth factor (bFGF) or nerve growth factor (NGF) (Rafiee et al., 2020; Lott et al., 2023; Zheng et al., 2021). Moreover, some researchers have shown that hypoxia can to modulate DPSCs differentiation. Indeed, Delle Monache et al. have shown that hypoxia, under certain *in vitro* conditions, can commit DPSCs towards a neuron-like phenotype and stimulate the secretion of growth factors known to stimulate neural differentiation of DPSCs (Delle Monache et al., 2022). In fact, conditioned media (CMs) taken from DPSCs cultured under hypoxic conditions have been shown to induce neural commitment in control DPSCs and the SHSY5Y human neuroblastoma-derived neural cell line (Zheng et al., 2021). For this reason, both DPSCs and their CMs represent potential tools to be exploited in therapeutic strategies for neurodegenerative diseases, as CMs can induce differentiation while avoiding the immune-related complications that might arise from direct cell transplantation (Delle Monache et al., 2022; Ogata et al., 2022).

Despite the growing interest in the neurogenic potential of DPSCs, the metabolic mechanisms underlying their neural commitment are not well understood. In particular, metabolomics studies describing how central metabolic pathways are remodelled during the transition from a mesenchymal to a neural phenotype of human DPSCs are lacking. Addressing this gap is critical, as metabolic reprogramming is increasingly recognized not merely as a consequence of differentiation, but as an active regulator of cell fate decisions. A deeper understanding of these metabolic shifts may therefore provide a rational basis for refining and optimizing differentiation protocols and for identifying metabolic markers associated with successful neural commitment. In this study, we used ^1^H NMR-based metabolomics to characterize dynamic metabolic changes during the neural commitment of human DPSCs. We analyzed metabolic profiles after 14 days of differentiation to investigate metabolomics changes occurring during neural commitment. Integrating metabolomics data with established neural commitment markers provides new insight into the metabolic reprogramming that accompanies DPSC neurogenesis and identifies candidate metabolic signatures associated with neural differentiation.

## 2 Materials and methods

### 2.1 Cell culture

DPSCs were purchased from Lonza (Walkersville, USA). The donor of DPSC was a 17-year-old male. As reported in the data sheet, the manufacturer tested the DPSCs for CD105, CD166, CD29, CD90, and CD73 antigens, showing a positivity rate greater than or equal to 90%. The cells were cultured in Dental Pulp Stem Cell BulletKit™ Medium which includes both basal medium and the necessary supplements for human dental pulp mesenchymal stem cell proliferation (Lonza, Walkersville, USA), in a humified incubator in a 5% CO_2_ atmosphere at 37 °C. The culture medium was replaced every 3 days and when 90% confluence was achieved, cells were harvested using 0.05% Trypsin-EDTA (Euroclone, Milan, Italy). Cells were cultured between P4-P8 for subsequent experiments.

### 2.2 *In vitro* DPSC neural commitment

To induce neural commitment, DPSCs were cultured for 14 days in Neurobasal A medium (neuro-induced DPSCs) supplemented with L-Glutamine, supplemented with B27 (Life Technologies, Monza, Italy), bFGF 40 ng/mL, and EGF 20 ng/mL (PeproThec, DBA, Milan, Italy). The induction media was replaced every 3 days. Morphological and flow cytometric analyses of CD44, CD90, CD105, CD73, STRO-1, CD14, CD19, B3-Tubulin, NFH, and GAP43 expression in untreated or EGF/bFGF-treated hDPSCs for 14 days were performed as previously described (Martellucci et al., 2019a) and in Supplemental Materials. A trypan blue assay were performed for metabolomics experiments to evaluate vitality.

### 2.3 Metabolomics based ^1^H NMR spectroscopy

Reagents: deuterated reagents (methanol (CD_3_OD), chloroform (CDCl_3_), deuterium oxide (D_2_O) and tetramethylsilane (TMS) were purchased from Cambridge Isotope Laboratories, Inc.; 3-(trimethylsilyl) propionic-2,2,3,3-d4 acid sodium salt (TSP) was obtained from Merck & Co, Montreal, Canada). Aqueous and organic metabolites were extracted from control and neuro-induced DPSCs, treated according to the protocol previously described (Santilli et al., 2023). Briefly, extraction of intracellular metabolites was performed by using a dual-phase extraction method (methanol/chloroform/water 1:1:1) on cell pellets. The polar phase containing water-soluble cellular metabolites was lyophilized while the organic fraction (lipid phase) was dried under nitrogen gas flow. Both phases of cell extracts were stored at −20 °C. The aqueous fraction from cells and extracellular medium samples (300 μL) were reconstituted in 700 μL D_2_O using -(trimethylsilyl) propionic-2,2,3,3-d4 acid sodium salt (TSP) (0.1 mM) as NMR internal standard. The organic fraction was reconstituted in 750 μL CDCl3: CD3OD (2:1) using tetramethylsilane (TMS) as internal chemical shift standard. High-resolution ^1^H NMR analyses were performed at 25 °C at 14.1 T and 9.4 T Bruker AVANCE spectrometers (Karlsruhe, Germany, Europe) on aqueous cell extracts. For each sample, a standard one-dimensional ^1^H NMR spectrum (NOESYPR1D) was acquired using the following parameters: relaxation delay of 5 s, 256 transients, mixing time of 100 ms, acquisition time of 1.39 s, spectral width of 20.83 Hz, and 32k complex data points. The one-dimensional spectra were processed using an exponential line-broadening function of 0.5 Hz, followed by baseline correction and manual phase adjustment. Chemical shifts were referenced to TSP at δ = 0.00 ppm. (Rossi et al., 2024). In particular, the quantification of aqueous metabolites, determined by comparing the integral of each metabolite to the integral of reference standard TSP and corrected by respective proton numbers for metabolite and TSP. Absolute quantification of individual aqueous metabolites was obtained from peak areas using correction factors determined by experiments at the equilibrium of magnetization (90) pulses, 40.00-s interpulse delay. The metabolite concentration was expressed as nmoles/10^6^ cells and then converted into metabolite percentage (relative to total metabolites evaluated in each sample; (% Met/Σ all Met).

To reduce potential variability related to differences in cell number and cell volume, relative lipid quantification was expressed as the percentage contribution of each individual integral to the total integral of all analyzed lipid signals. For exploratory visualization purposes, Volcano plot analysis in MetaboAnalyst was generated using an unpaired parametric t-test with unadjusted p-values. Multivariate principal component analyses (PCA) were performed using the MetaboAnalyst 6.0 tool to visualize the relationship between sample groups and metabolic profiles. PCA was performed after log transformation and Pareto scaling. PERMANOVA (Permutational Multivariate Analysis of Variance) using the Euclidean distance based on the PCs was used to assess overall metabolomic differences between groups based on distance matrices, using 999 permutations. Metaboanalyst 6.0 was also used to identify the biochemical pathways in neural differentiated cells determined by Pathways impact (a parameter which identify top-level altered pathways, as described in www.metaboanalyst.ca) and quantitative enrichment analyses. Pathway impact reflected the deviation of metabolic pathways between the two groups based on the metabolite concentration. Quantitative enrichment analysis is a pathway analysis method that allows the identification of biologically meaningful patterns that are significantly enriched, and therefore systemically altered, according to quantitative metabolomic data.

Data were analyzed using GraphPad Software version 3.03. Statistical significance of differences was determined by non-parametric Mann-Whitney U test. Differences were considered significant at P < 0.05.

### 2.4 Lipid analysis by gas chromatography

Lipids were transmethylated with boron trifluoride-methanol solution (Sigma-Aldrich, Burlington, MA, USA) and methanolic NaOH (Supelco, Bellefonte, PA) for 60′ at 90 °C. Fatty acid methyl esters were extracted with hexane and analyzed by gas chromatography (GC system 7890 Agilent Technologies, Palo Alto, CA). The gas chromatograph was equipped with a fused silica capillary column (Supelcowax, 30 m 0.53 mm i.d. and 1.0 μm film thickness, Supelco) and a flame ionization detector. The injector (split 25:1) temperature was 260 °C, and the detector temperature was set at 260 °C. The heating program began at 210 °C, increased by 2 °C per minute and was held at 240 °C for 10 min. The carrier gas was helium at a flow rate of 20 mL/min. The fatty acid methyl esters (ME) were identified by comparison with PUFA II authentic standards (Supelco) and calculated as percentages of total FA.

## 3 Results

### 3.1 Changes in neural markers following neural commitment from DPSCs

**FIGURE 1:**
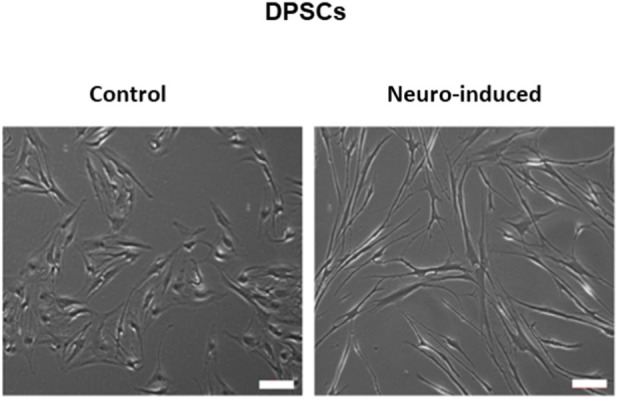
Representative images showing the morphological differences between untreated DPSCs and DPSCs treated with EGF and bFGF for 14 days. Bar 100 µM.

Control DPSCs exhibit a morphology typical of mesenchymal stem cells, characterized by a spindle-shaped appearance. In contrast, neural commitment of DPSCs determined a more elongated, neuron-like morphology, confirming that the differentiation protocol induced a shift towards a neuron-like phenotype (Figure 1). As specified in our previous article, the change in stem cell morphology was associated with a change in mesenchymal and neural markers after 14 days of EGF/FGF (Martellucci et al., 2019a). Control DPSCs showed high expression of mesenchymal stem cell markers, while exhibiting low expression of neural markers, in keeping with their mesenchymal phenotype. In contrast, neuro-induced DPSCs show a marked reduction in stem cell markers expression but high expression of neural markers (β3-Tubulin, NFH, GAP43) in flow cytometry analysis (Martellucci et al., 2019a).

### 3.2 Change in metabolome profile following neural commitment from DPSCs

**FIGURE 2:**
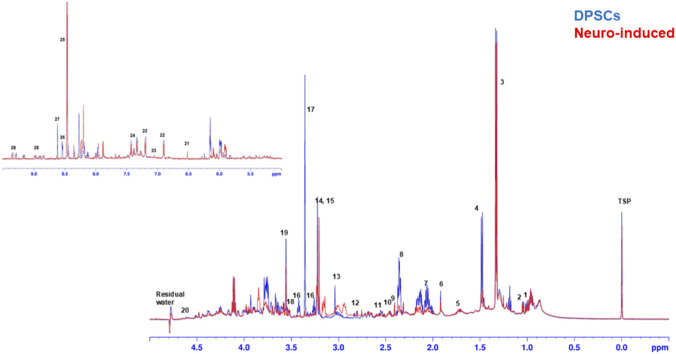
Representative ^1^H NMR spectra (14.1T) from aqueous intracellular extract (endometabolome) of control DPSCs and neuro-induced cells with peak assignment: 1: isoleucine; 2: valine; 3: lactic acid; 4: alanine; 5: lysine; 6: acetic acid; 7: glutamate + glutamine + glutathione; 8: glutamate; 9: pyruvic acid; 10: succinic acid; 11: glutathione; 12: aspartic acid; 13: total creatine; 14: choline; 15: phosphocholine; 16: taurine; 17: scyllo-inositol; 18: myo-inositol; 19: glycine; 20: glucose; 21: fumaric acid; 22: tyrosine; 23: histidine; 24: phenylalanine; 25: formic acid; 26: ATP + ADP; 27: AMP; 28: NAD; internal standard -(trimethylsilyl) propionic-2,2,3,3-d4 acid sodium salt (TSP). Note that the down-field spectral region (6.6–9.5 ppm) has been amplified 6x respect to the up-field spectral region (0.5–4.5 ppm).

Representative ^1^H NMR spectra of the aqueous endometabolome of control DPSCs and neuro-induced cells, including peak assignment are shown in Figure 2.

To gain insight into how the neural differentiation alters the cellular metabolism, ^1^H NMR metabolomics analysis was performed on cell extracts (polar and organic fractions). The NMR detected alterations reflect the metabolic state of the cell and its possible modulation due to DPSC differentiation. Overall, 30 metabolites were quantified including several amino acids, organic acids, nucleotides, sugars, among others (Table 1; Supplementary Table S1).

**TABLE 1:** Relative quantification of metabolites (% Met/Σ all Met) detected by ^1^H NMR (14.1 T) in aqueous extracts of Control or Neuro-induced DPSCs. Metabolites are grouped according to functional pathways.

Metabolic Pathway	ID*	Metabolite	Control DPSCs (n = 6)	Neuro-induced DPSCs (n = 4)
Amino acid metabolism	2	**Valine**	1.55 ± 0.59	1.89 ± 0.23
	4	**Alanine**	**5.91 ± 1.13**	**4.16 ± 0.28**
	5	**Lysine**	4.42 ± 2.42	5.58 ± 1. 01
	7	**Glx**	21.11 ± 5.28	20.32 ± 2.85
	8	**Glutamic acid**	**7.64 ± 2.34**	**3.90 ± 0.96**
	12	**Aspartic acid**	**1.67 ± 0.34**	**3.82 ± 1.33**
	19	**Glycine**	2.85 ± 1.06	3.75 ± 0.18
	22	**Tyrosine**	1.41 ± 0.30	1.97 ± 0.23
	23	**Histidine**	0.32 ± 0.20	0.55 ± 0.34
	24	**Phenylalanine**	3.79 ± 1.74	6.10 ± 1.30
	1	**Isoleucine**	1.18 ± 0.32	1.34 ± 0.16
Glycolysis and TCA cycle	3	**Lactic acid**	16.06 ± 0.3.12	22.14 ± 8.04
	21	**Fumaric acid**	0.07 ± 0.06	0.11 ± 0.07
	10	**Succinic acid**	0.51 ± 0.36	0.40 ± 0.14
	20	**Glucose**	0.56 ± 0.34	1.07 ± 0.53
	9	**Pyruvic acid**	0.63 ± 0.18	0.42 ± 0.10
	6	**Acetic acid**	1.59 ± 1.29	2.18 ± 1.74
Redox metabolism	11	**Glutathione**	2.40 ± 0.82	2.95 ± 0.88
	16	**Taurine**	1.86 ± 0.58	1.31 ± 0.17
High-energy nitrogenous metabolites	13	**Creatine plus Phosphocreatine**	1.19 ± 0.35	1.19 ± 0.45
Pospholipid metabolism	14	**Free choline**	0.80 ± 0.60	1.33 ± 0.22
	15	**Phosphocholine**	**0.73 ± 0.59**	**1.41 ± 0.70**
	18	**Myo-inositol**	4.16 ± 3.21	1.42 ± 1.77
	17	**Scyllo-inositol**	0.27 ± 0.09	0.17 ± 0.06
One-carbon metabolism	25	**Formic acid**	8.33 ± 9.39	2.09 ± 1.54
Nucleotide and energy metabolism	26	**ATP + ADP**	0.28 ± 0.57	0.30 ± 0.37
	27	**AMP**	0.38 ± 0.44	0.12 ± 0.17
	28	**NAD**	0.20 ± 0.28	0.35 ± 0.40

In bold are reported the statistical significance between DPSCs and neuro-induced DPSC cells ad determined by Mann-Whitney test (p < 0.05), *Peak assignment as in Figure 2. The peak n7 shown in Figure 2 is *a* pool *of*: glutamate + glutamine + glutathione (Glx).

**FIGURE 3:**
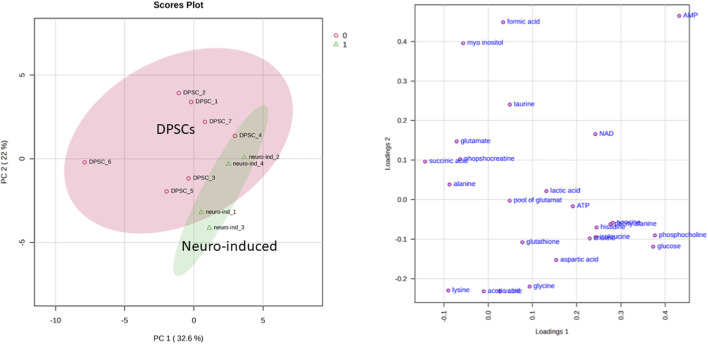
2D Score plot (left panel) and loading plot (right panel) of the Principal Component Analysis (PCA) analysis for aqueous metabolome of DPSCs and neuro-induced cells.

Exploratory unsupervised PCA (Figure 3) was performed on the aqueous metabolome of DPSCs (n = 7) and neuro-induced (n = 4) cells. The first two principal components accounted for 54.6% of the total variance and showed a partial separation between DPSC sand neuro-induced DPSC samples. This group separation was supported by PERMANOVA analysis (F = 3.53, R^2^ = 0.28, p = 0.027 based on 999 permutations). The corresponding loading plots suggested that relative differences in phosphocholine (PCho), glucose, AMP, and several amino acids contributed to the variation captured by PC1, whereas myo-inositol, alanine, and lysine mainly contributed to PC2.

Given the partial separation between groups observed in the multivariate PCA analysis, univariate statistical tests were performed to assess statistically significant differences in metabolite concentrations between DPSCs and neuro-induced DPSC cells.

**FIGURE 4:**
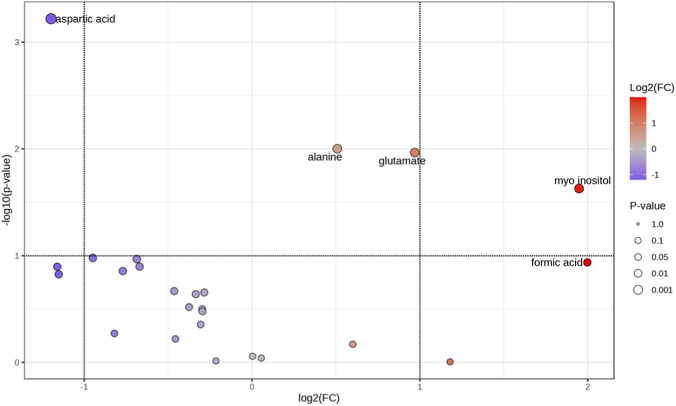
Important features selected by volcano plot with fold change threshold: 2.0 (x-axis) and P value threshold: 0.1 (y-axis).

Volcano plot analysis (Figure 4), integrating fold change (Supplementary Table S2) and univariate statistical significance, identified a subset of metabolites, including aspartic acid, myo-inositol, and phosphocholine, that exhibited both substantial changes in abundance and statistically significant differences (p < 0.05), highlighting potential metabolites associated with the neuro-induction process.

**FIGURE 5:**
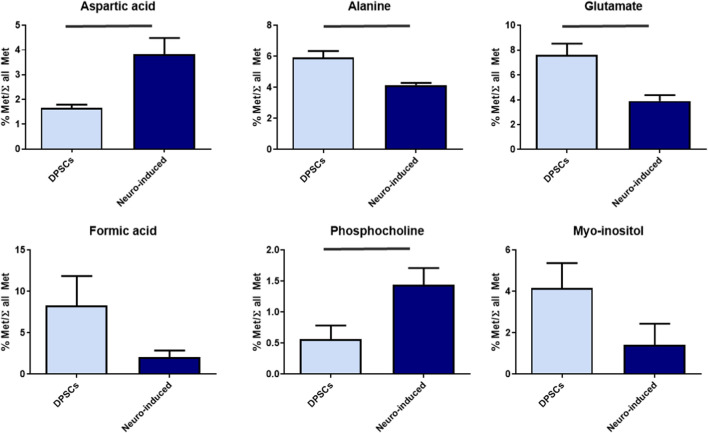
Relative quantification of significant aqueous metabolites in DPSCs (n = 7) and neuro-induced cells (n = 4).

Consistently, non-parametric Mann-Whitney U test (Figure 5) revealed that neuro-induced DPSCs exhibited significantly higher levels of aspartate (p = 0.006) and phosphocholine (p = 0.032), whereas undifferentiated DPSCs showed higher concentrations of alanine (p = 0.012), glutamate (p = 0.042), and although non statistical myo-inositol (p = 0.18) and formic acid (p = 0.16).

**FIGURE 6:**
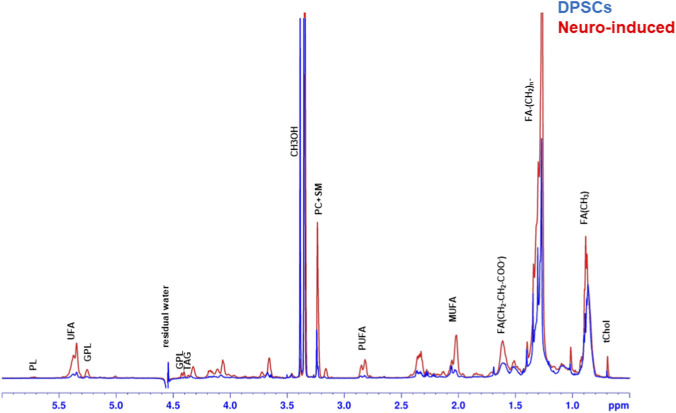
Representative ^1^H NMR spectra (14.1T) from aqueous intracellular extract (endometabolome) of control DPSCs and neuro-induced cells with peak assignment: Peak assignment in organic fraction: signals of the pool of acyl chains (FA) determined at 0.9 ppm (ω-CH_3_ of FA), at 1.56 ppm (-CH_2_-CH_2_-COO^-^ of FA); glycerophospholipids (GPL); residual water HOD; methanol (MeOH); total cholesterol (tChol); monounsaturated fat acids (MUFA); plasmalogens (PL); polyunsaturated FA (PUFA); sphingomyelin (SM); unsaturated FA (UFA); phosphatidylcholine and lysophosphatidylcholine(PC) and triacyclglycerols (TAG). In bold the resonance of FA.

**FIGURE 7:**
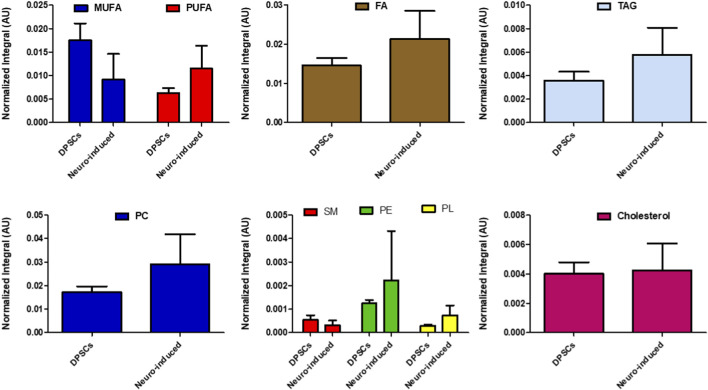
Relative quantification of the main class of lipids (% Met/Σ all Met) in organic extracts of control and neuro-induced DPSCs (n = 2). Fatty acid (FA); monounsaturated FA (MUFA); phosphatidylcholine (PC); phosphatidylethanolamine (PE); phospholipids (PL); polyunsaturated FA (PUFA); sphingomyelin (SM); triacylglycerols (TAG).

Representative spectra of the lipid samples are reported in Figure 6. We observed an overall increase in fatty acids, triacylglycerols, and phospholipids in neuro-induced DPSCs as compared to control cells (Figure 7). Although it is tempting to speculate that neural differentiation commitment was associated with an overall change in lipid content, given the limited number of biological replicates (n = 2), out findings should be considered exploratory and descriptive and require further explorations.

An increase in lipid unsaturation was observed, with the PUFA/MUFA ratio increasing by approximately 30%, from 3.6 in undifferentiated DPSCs to 4.8 in neuro-induced DPSCs, as determined by ^1^H NMR analysis of the lipid fraction.

Gas chromatography analysis of total fatty acids in basal DPSCs (Supplementary Table S3) revealed a fatty acid profile dominated by saturated fatty acids, mainly palmitic acid (C16:0) and stearic acid (C18:0), and an overall higher proportion of monounsaturated fatty acids compared to polyunsaturated fatty acids.

#### 3.2.1 Multivariate analyses and enrichment pathways after neural commitment from DPSCs

**FIGURE 8:**
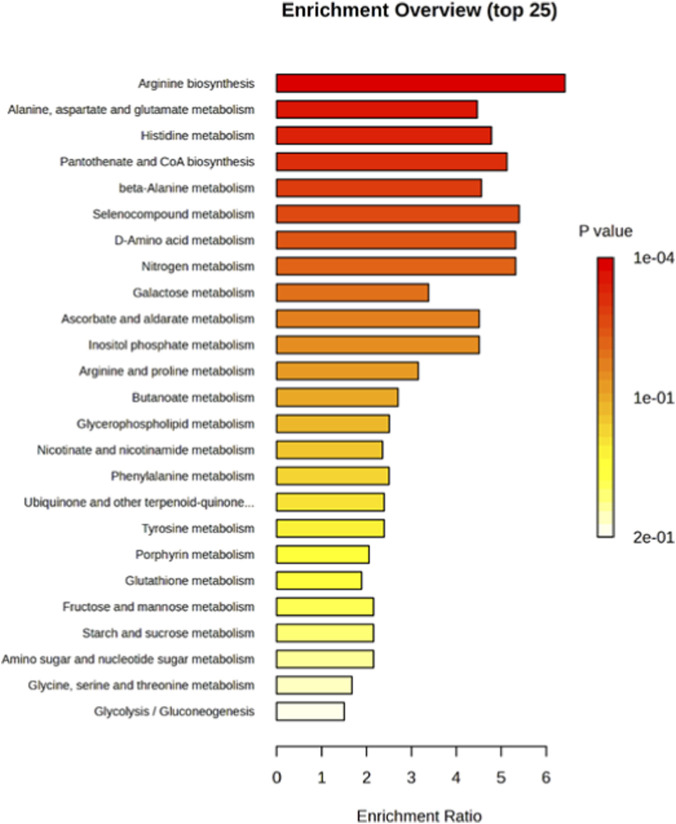
Enrichment Analysis of significantly altered metabolites of control and neuro-induced DPSCs. The most impacted pathways (p-values <0.05) are shown in red.

Metabolite Set Enrichment Analysis (MSEA) is a powerful approach for identifying biologically meaningful patterns that are significantly enriched within quantitative metabolomic datasets. In our analysis, the significantly altered metabolites were mapped to several metabolic pathways potentially impacted by neural commitment. Notably, quantitative enrichment analysis revealed that amino acids metabolism such as alanine, aspartate, and glutamate metabolism, were among the most enriched pathways associated with the neurodifferentiation process (Figure 8).

## 4 Discussion

Neural differentiation represents a highly coordinated process involving extensive transcriptional, morphological, and metabolic remodelling. Neural stem and progenitor cells (NSPCs) typically rely on glycolysis to sustain rapid proliferation and provide biosynthetic precursors. As differentiation progresses, these cells undergo a gradual metabolic transition toward mitochondrial oxidative phosphorylation (OXPHOS), a shift that enables mature neurons to meet the substantial ATP demands required for membrane excitability, synaptic transmission, and ion homeostasis. This metabolic remodelling is orchestrated by key regulators such as AMP-activated protein kinase (AMPK), the mammalian target of rapamycin (mTOR), and PGC-1α, which collectively promote mitochondrial biogenesis and function (Agostini et al., 2016; Zheng et al., 2016; Yan et al., 2021; Hu et al., 2016; Wanet et al. 2015). While mitochondrial maturation is a well-recognized feature of neural development, the metabolic reprogramming associated with neural commitment remains incompletely defined in dental pulp stem cells.

In the present study, ^1^H NMR metabolomics revealed alterations in intracellular amino acid and phospholipid metabolism following a 14-day neural induction protocol. Although steady-state metabolite levels do not directly inform on metabolic fluxes, the observed differences indicate a reorganization of intracellular metabolic pools during neural commitment. The moderate separation observed in the principal component analysis (PCA) could be consistent with a partial metabolic divergence during the early stages of neural differentiation rather than with a complete metabolic transformation, which would be expected in more advanced stages of maturation. Neuro-induced DPSCs exhibited elevated levels of aspartate and phosphocholine. Beyond its canonical role as an amino acid intermediate, aspartate functions as a central metabolic node linking the malate-aspartate shuttle, nucleotide biosynthesis, and TCA cycle anaplerosis. Because intracellular aspartate availability is coupled to mitochondrial activity and redox balance, its elevation during DPSC neural induction could reflect enhanced mitochondrial-cytosolic coupling and biosynthetic competence associated with lineage progression. In addition, as mitochondrial metabolism modulates metabolite-dependent chromatin remodeling, increased aspartate levels may be consistent with a metabolic state permissive for neural transcriptional reprogramming. Although mechanistic validation is required, these findings support the interpretation that aspartate enrichment could represent an integrated metabolic adaptation accompanying neural commitment rather than a passive by product of differentiation (Qi et al., 2021; Rae et al., 2025). Phosphocholine levels were significantly increased following neural induction. As an intermediate of the CDP-choline (Kennedy) pathway, phosphocholine directly fuels phosphatidylcholine synthesis. Enhanced phosphocholine availability may therefore reflect activation of membrane biogenesis programs required for neurite extension and cytoskeletal remodelling during neural differentiation (Marcucci et al., 2010). Beyond energy metabolism, alterations in lipid profiles were observed in neuro-induced DPSCs, although at exploratory level. The enrichment of PUFAs, particularly docosahexaenoic acid (DHA), is consistent with neural membrane specialization, influencing fluidity, vesicle trafficking, and the functionality of membrane-associated receptors and ion channels (Dec et al., 2023). In particular, the ratio of n-6 to n-3 PUFAs is known to influence neural differentiation efficiency and electrophysiological maturation, with an excess of n-6 fatty acids negatively impacting synaptic network formation and gene expression (Dec et al., 2023). An increased relative level of triacylglycerols was also observed. Although the present data are limited (n = 2), this pattern could reflect shifts in lipid handling during differentiation and may be related to the balance between energy storage and membrane lipid synthesis. Such lipid dynamics are intimately linked to mitochondrial membrane plasticity, which modulates both bioenergetic capacity and epigenetic signaling pathways (Ramosaj et al., 2021). Recent findings have further underscored the regulatory role of lipid droplets (LDs) in stem cell differentiation. Plin2-mediated LD mobilization has been shown to coordinate phospholipid homeostasis, mitochondrial function, and histone acetylation-processes central to the transition from pluripotency to lineage specification (Wu et al., 2022). Although these mechanisms were not directly assessed in the present study, the lipid remodeling observed in neuro-induced DPSCs is compatible with such interactions between lipid metabolism, mitochondrial activity, and epigenetic control (Ramosaj et al., 2021). These mechanisms were not directly assessed in the present study. Therefore, the lipid-related changes described here should be considered preliminary and hypothesis-generating. Nevertheless, the observed trends are consistent with previously reported membrane adaptations occurring during neural differentiation (Qi et al., 2021; Rae et al., 2025) and warrant validation in larger experimental cohorts.

Of note, undifferentiated DPSCs displayed higher concentrations of myo-inositol, alanine, and glutamate. Myo-inositol serves as the structural backbone for phosphoinositide synthesis, thereby sustaining PI3K/AKT-dependent signaling and membrane-associated second messenger cascades that are tightly linked to stem cell maintenance and proliferative competence. Elevated alanine levels may reflect enhanced pyruvate transamination via alanine aminotransferase, a mechanism that couples glycolytic flux to nitrogen shuttling and redox balance while facilitating metabolic flexibility under high proliferative demand. Similarly, higher glutamate abundance is commonly associated with glutaminolysis and transamination reactions contributing to TCA cycle intermediate availability. While these interpretations are biologically plausible, it is important to emphasize that steady-state ^1^H NMR metabolomics measures relative intracellular metabolite pools and does not directly assess metabolic fluxes or bioenergetic activity. Therefore, the proposed metabolic shift should be considered associative rather than a direct demonstration of functional metabolic reprogramming.

Together, these findings delineate a metabolic landscape that evolves during neural induction, with increased involvement of mitochondrial-associated pathways, without directly demonstrating a functional transition from glycolytic to oxidative metabolism. Although our metabolomic data are correlative and do not capture metabolic fluxes, the observed metabolite patterns are consistent with previous reports describing metabolic remodelling during stem cell differentiation and neural maturation.

A limitation of our study is that ^1^H NMR metabolomics provides snapshots of intracellular metabolite pools and does not directly measure metabolic fluxes or extracellular secretion. Additionally, the 14-day differentiation protocol likely captures cells in an intermediate neuron-like state rather than fully mature neurons. Future studies integrating flux analysis, mitochondrial activity assays, and longer differentiation time points could further elucidate the temporal dynamics of metabolic reprogramming during DPSC neurogenesis. Despite its exploratory nature, this study provides an integrated analysis of both polar and lipid intracellular metabolomes in a human DPSC neural commitment model, contributing to the quantitative biochemical context to phenotypic differentiation processes that are typically described at transcriptomic or morphological levels (Gul et al., 2025; Alqahtani et al., 2025; Pardo-Rodríguez et al., 2025).

## 5 Conclusion

In summary, our data indicate that DPSC neural commitment is accompanied by measurable changes in intracellular polar and lipid metabolite profiles. These variations suggest alterations in metabolic pathways and lipid composition occurring during the differentiation process. While steady-state ^1^H NMR metabolomics does not allow direct assessment of metabolic fluxes or functional bioenergetic transitions, the observed metabolite patterns are compatible with previously described metabolic adaptations associated with neural differentiation. Overall, this study provides a descriptive and exploratory metabolic characterization of DPSC neural commitment and highlights candidate metabolic signatures that may be relevant to this process. Further investigations integrating functional and flux-based approaches will be necessary to clarify the extent and functional significance of these metabolic changes.

## Data Availability

The original contributions presented in the study are included in the article/Supplementary Material, further inquiries can be directed to the corresponding authors.
