# Supplementary material for: NMR metabolomics assessment of neural commitment of human dental pulp-derived stem cells
Source: Front Mol Biosci. 2026 May 22;13:1735166. doi: 10.3389/fmolb.2026.1735166 (PMC13236527; doi:10.3389/fmolb.2026.1735166)
Supplement: Supplementary file 1 [file Table1.docx]

**NMR metabolomics assessment of neural differentiation commitment of human dental pulp-derived stem cells**

Mattea Chirico^1^†, Maria Elena Pisanu^1^†, Emanuela Mari^2^, Valeria Manganelli^3^, Fanny Pulcini^4^, Loreto Lancia^4^, Rita Di Benedetto^5^, Simona Delle Monache^4^, Egidio Iorio^1^*, Vincenzo Mattei^2^*

**Methods:**

Flow Cytometry of DPSC Neural Commitment

Flow cytometric analysis of CD44, CD90, CD105, CD73, STRO-1, CD14, CD19, β3-Tubulin, NFH, and GAP43 expression in untreated or EGF/bFGF-treated hDPSCs (14 days) were performed as previously described (Martellucci S, Santacroce C, Manganelli V, et al. Isolation, Propagation, and Prion Protein Expression During Neuronal Differentiation of Human Dental Pulp Stem Cells.. Isolation, Propagation, and Prion Protein Expression During Neuronal Differentiation of Human Dental Pulp Stem Cells. J Vis Exp. (2019) 145:10.3791/59282. doi:10.3791/59282).

All flow cytometry data were generated from at least three independent biological replicates, and for each biological replicate, technical replicates were acquired.

The gating strategy was standardized as follows. Histograms represent the logarithm of fluorescence versus cell number, based on the cell population of a side scatter/forward scatter (SS/FS) histogram. Debris, (all of which should be FSC-low), are removed from the analysis by setting and adjusting the FSC Threshold as necessary.

**Table S1**. ^1^H NMR assignment (14.1 T) of aqueous metabolites identified. in DPSCs and neural differentiated cells, listed by chemical shift value. Multiplicity: s, singlet; d, doublet; dd, doublet of doublets; ddd, doublet of doublets of doublets; t, triplet; q, quartet; m, multiplet; br, broad signal. In bold, resonance (ppm) used for quantification.

| **Metabolite** | **HMDB ID, KEGG ID** | ***δ* ^1^H in ppm (multiplicity, assignment)** |
| --- | --- | --- |
| **Acetic acid** | HMDB0000042, C00033 | **1.92** (s, *β-*C**H_3_**) |
| **ADP** | HMDB0001341, C00008 | 4.24 (m, 5’-C**H_2_** ribose), 4.39 (m, 4’-C**H** ribose), 4.62 (m, 3’-C**H** ribose), 6.15 (d, 1’-C**H** ribose), 8.28 (s, 2-C**H** ring), **8.54** (s, 8-C**H** ring) |
| **Alanine** | HMDB0000161, C00041 | **1.48** (d, *β-*C**H_3_**), 3.78 (q, *α-*C**H**) |
| **AMP** | HMDB0000045, C00020 | 4.02 (m, 5’-C**H_2_** ribose), 4.51 (m, 3’-C**H** ribose),6.15 (d, 1’-C**H** ribose), 8.28 (s, 2-C**H** ring), **8.62** (s, 8-C**H** ring) |
| **Aspartic acid** | HMDB0000191, C00049 | **2.68/2.82** (dd/dd, *β-*C**H_2_**), 3.90 (dd, *α-*C**H**) |
| **ATP** | HMDB0000538, C00002 | 4.22/4.29 (m/m, 5’-C**H_2_** ribose), 4.41 (m, 4’-C**H** ribose), 4.62 (m,  3’-C**H** ribose), 6.15 (d, 1’-C**H** ribose), 8.28 (s, 2-C**H** ring), **8.55** (s, 8-C**H** ring) |
| **Choline** | HMDB0000097, C00114 | **3.21** (s, N(C**H_3_**)_3_), 3.53 (m, N-C**H_2_**), 4.07 (m, C**H_2_**-OH) |
| **Creatine** | HMDB0000064, C00300 | **3.04** (s, N-C**H_3_**), 3.93 (s, N-C**H_2_**) |
| **Formate** | HMDB0000142, C00058 | **8.46** (s, HO-**H**C=O) |
| ***α*-Glucose** | HMDB0003345, C00031 | **5.24** (d, 1-C**H**) |
| ***β*-Glucose** | HMDB0000122, C00221 | **4.65** (d, 1-C**H**) |
| **Glutamate** | HMDB0000148, C00025 | 2.05/2.14 (m/m, *β-*C**H_2_**), **2.35** (m, *γ-*C**H**_2_),  3.76 (dd, *α-*C**H**) |
| **Glutamine** | HMDB0000641, C00064 | 2.14 (m, *β-*C**H_2_**), **2.46** (m, *γ-*C**H_2_**), 3.78 (t, *α-*C**H**) |
| **Glutathione** | HMDB0000125, C00051 | 2.15 (m, *β*-C**H_2_** Glu), **2.55** (m, *γ*-C**H_2_** Glu), 2.96 (m, *β*-C**H_2_** Cys), 3.78 (m, *α*-C**H** Glu & *α*-C**H_2_**, Gly), 4.56 (dd, C**H**) |
| **Glycerophosphocholine** | HMDB0000086, C00670 | **3.24** (s, N(C**H_3_**)_3_) |
| **Glycine** | HMDB0000123, C00037 | **3.56** (s, α-C**H_2_**) |
| **Histidine** | HMDB0000177, C00135 | **7.08** (s, 5-C**H** ring), 7.84 (s, 2-C**H**) |
| **Isoleucine** | HMDB0000172, C00407 | 0.94 (t, *δ*-C**H_3_**), **1.02** (d, *γ’*-C**H_3_**), 1.28/1.45 (m/m, *γ*-C**H_2_**),  1.98 (m, *β*-C**H**), 3.66 (d, *α*-C**H**) |
| **Lactic acid** | HMDB0000190, C00186 | **1.33** (d, C**H_3_**), 4.11 (q, C**H**) |
| **Lysine** | HMDB0000182, C00047 | 1.48 (m, *γ*-C**H_2_**), **1.72** (m, *δ*-C**H_2_**), 1.91 (m, *β*-C**H_2_**),  3.03 (t, *ε*-C**H_2_**, t), 3.77 (t, *α*-C**H**) |
| ***myo*-inositol** | HMDB0000211, C00137 | 3.28 (t, 5-C**H**), **3.54** (dd, 1-C**H** & 3-C**H**),  3. 63 (t, 4-C**H** & 6-C**H**), 4.07 (t, 2-C**H**) |
| **NAD^+^** | HMDB0000902, C00003 | 6.03 (d, 1’-C**H** ribose-adenine), 6.11 (d, 1’-C**H** ribosenicotinamide), 8.18 (s, 2-C**H** adenine), 8.20 (m, 5-C**H** nicotinamide), 8.43 (s, 8-C**H** adenine), 8.84 (d, 4-C**H** nicotinamide), **9.15** (d, 6-C**H** nicotinamide), 9.34 (s, 2-C**H** nicotinamide) |
| **Phenylalanine** | HMDB0000159, C00079 | **7.34** (m, 2-C**H** & 6-C**H** ring), 7.39 (m, 4-C**H** ring),  7.43 (m, 3-C**H** & 5-C**H** ring) |
| **Phosphocholine** | HMDB0001565, C00588 | **3.23** (s, N(C**H_3_**)_3_), 3.60 (m, N-C**H_2_**), 4.17 (m, HPO4^―^-C**H_2_**) |
| **Phosphocreatine** | HMDB0001511, C02305 | **3.05** (s, N-C**H_3_**), 3.95 (s, N-C**H_2_**) |
| **Pyruvyc acid** | HMDB0000243, C00022 | **2.38** (s, C**H_3_**) |
| **Scyllo-inositol** | HMDB0000160  C06153 | **3.35** (s, C**H**) |
| **Succinic acid** | HMDB0000254,  C00042 | **2.41** (s, C**H_2_**) |
| **Taurine** | HMDB0000251,  C00245 | 3.27 (t, S-C**H_2_**), **3.42** (t, N-C**H_2_**) |
| **Threonine** | HMDB0000167,  C00188 | **1.33** (d, *γ*-C**H_3_**), 3.59 (d, *β*-C**H)**, 4.25 (dd, *α*-C**H**) |
| **Tyrosine** | HMDB0000158,  C00082 | **6.91** (d, 3-C**H** & 5-**H** ring), 7.20 (d, 2-C**H** & 6-**H** ring) |
| **UDP-GalNAc** | HMDB0000304, G10611 | **5.55** (dd, 1’’-C**H** galactose) |
| **UDP-GlcNAc** | HMDB0000290, C00043 | 2.08 (s, C**H_3_** NAc), 3.82 (m, 3’’-C**H** glucose & 6´´-C**H_2_** glucose), 3.88 (m, 6’’ C**H_2_** glucose), 4.00 (m, 2’’-C**H** glucose), 4.19/4.26 (m/m, 5’-C**H_2_** ribose), 4.30 (m, 4’-C**H** ribose), 4.38 (m, 2’-C**H** ribose & 3’-C**H** ribose), 5.52 (dd, 1’’-C**H** glucose), **5.99** (m, 5C**H** & 1’-C**H** ribose), 7.96 (d, 6-C**H**) |
| **Valine** | HMDB0000883,  C00183 | 1.00 (d, *γ’*-C**H_3_**), **1.05** (d, *γ-*C**H_3_**), 2.27 (m, *β-*C**H**),  3.62 (d, *α*-C**H**) |

**Table S2**: Important features identified by fold changes analysis

|  | **Compounds** | **Fold Change** | **Log2 (FC)** |
| --- | --- | --- | --- |
|  | **formic acid** | 3.9935 | 1.9976 |
|  | **myo-inositol** | 3.8594 | 1.9484 |
|  | **aspartic acid** | 0.43608 | -1.1973 |
|  | **phosphocholine** | 0.4479 | -1.1601 |
|  | **glucose** | 0.45019 | -1.1514 |

**Table S3**: Relative quantification of fatty acid (FA) of control DPSCs cells (mean ± semidispersion; n=2). Fatty acid saturated (SFA) and unsaturated fatty acid: monounsaturated (MUFA) and polyunsaturated (PUFA). The left column reports the fatty acid composition (length and degree of unsaturation).

| **Fatty acid** | % FA methyl esters /total FA |
| --- | --- |
| **C16:0** | 22.58±2.79 |
| **C16:1** | 3.31±0.83 |
| **C18:0** | 15.71±1.08 |
| **C18:1** | 32.48±6.95 |
| **C18:2** | 4.57±0.27 |
| **C18:3** | 1.84±0.36 |
| **C20:3** | 1.90±1.10 |
| **C20:4** | 5.04±1.81 |
| **C20:5** | 0.59±0.02 |
| **C22:4** | 1.57±0.69 |
| **C22:5** | 2.66±1.01 |
| **C22:6** | 8.53±2.97 |
|  |  |
| **SFA** | 38.29±1.71 |
| **MUFA** | 35.80±2.32 |
| **PUFA** | 26.69±2.76 |
